# Assessing Knowledge of Community Pharmacists on Cancer: A Pilot Study in Ghana

**DOI:** 10.3389/fpubh.2019.00013

**Published:** 2019-02-01

**Authors:** Kofi Boamah Mensah, Varsha Bangalee, Frasia Oosthuizen

**Affiliations:** ^1^Directorate of Oncology, Komfo Anokye Teaching Hospital, Kumasi, Ghana; ^2^Discipline of Pharmaceutical Sciences, College of Health Sciences, University of KwaZulu-Natal, Durban, South Africa

**Keywords:** cancer, knowledge, signs and symptoms, risk factors, community pharmacists, Ghana

## Abstract

**Background:** GLOBOCAN estimates that 16,600 cases of cancer occur annually in Ghana. Community pharmacists are the first point of contact to the public due to their accessibility, wide spread and credibility. They are often looked upon to provide first aid and treatment of common illness. They provide health information and support on diseases, e.g., cancer. Their role also extends to the patients' relatives. Thus, the level of knowledge and awareness of community pharmacists are of paramount importance in order to assure best healthcare advice is provided to the public. Goals of this pilot study were; (1) to collect a preliminary data on knowledge of risk factors, signs, and symptoms of cancer, (2) to ascertain the adequacy of the research survey in determining their level of knowledge, (3) to assess the viability of a full-scale study on community pharmacists.

**Methods:** A cross-sectional survey was conducted using a self-administered questionnaire to assess the knowledge of signs and symptoms and risk factors of cancer among 150 community pharmacists.

**Key Findings:** Score for knowledge on cancer among community pharmacists indicated that 76.7% had poor knowledge. Responses of community pharmacists toward a list of warning signs and symptom of cancer indicated poor level of knowledge (82%). Community pharmacists recorded poor level of knowledge (65.3%) on causes and risk factors for cancer. Correlation analysis shows that age has a relation with level of knowledge on signs and symptoms of cancer.

**Conclusion:** This pilot study provided a valuable data which indicated that community pharmacists in Ghana have poor level of knowledge on cancer. The findings obtained from the study agree with findings of other studies conducted in this area which suggest that survey instrument was adequate to assess the knowledge level of community pharmacist in Ghana. Though the response was low, data obtained indicate a viability and need of conducting a full-scale research in this workforce to get a better assessment of the level of knowledge of community pharmacists on cancer in Ghana.

## Introduction

Cancer is the second leading cause of death globally, and was responsible for 8.8 million deaths in 2015 (1). Globally, nearly 1 in 6 deaths is due to cancer and approximately 70% of deaths from cancer occurs in low- and middle-income countries (1). The incidence and mortality from cancer has been increasing in low and middle income countries (LMIC) compared to the western world despite advances in the research on cancer (2).

In Ghana, as in most developing countries, the burden of non-communicable diseases is increasing rapidly (3). GLOBOCAN estimates that 16,600 cases of cancer occur in Ghana annually (2). A study conducted at the Korle-Bu Teaching Hospital (KBTH), the biggest Teaching Hospital in Ghana, reviewed 3,659 cancer deaths over a 10-year period (4). Published data from Komfo Anokye Teaching Hospital (KATH), the second largest teaching hospital in Ghana, estimated that between 400 and 500 new cancer cases were diagnosed annually at the hospital from 2004 to 2006 (5).

In Ghana, there is low public awareness of cancer which results in late presentation of the disease at health facilities and hence poor treatment outcomes. This has contributed to increasing incidence and mortality of the disease as reported in LMIC (2).

Health care personnel are the main source of medical information to the general public and patients. This is due to their continual contact with patients and their relatives. Community pharmacists are the first point of contact to the public or community. This is often due to their accessibility, credibility (6, 7) and their wide spread within the public sector (8–11). They are often looked upon to provide symptoms assessment, treatments to minor illness, make necessary referrals, and providing life style advice. Their roles expand beyond patients to their circle of family and friends. It is thus important that the facts that they provide is correct and help in building awareness.

At present, the practice of pharmacy has shifted to include provision of patient-oriented pharmaceutical services in addition to the traditional service provision. Because of these important roles that community pharmacist's play, this workforce was chosen to be our target population for this pilot study. The aim of this pilot was to;

1. To collect a preliminary data on knowledge of community pharmacists on cancer, its risk factors, its signs and symptoms.
2. Ascertain the adequacy of the research survey in determining level of knowledge of community pharmacists on cancer, risk factors, signs and symptoms.
3. To assess the viability of a full-scale study on this workforce i.e., community pharmacists.

We report here the results of a pilot study which was the first step of a regional project aimed at assessing knowledge on cancer, causes and risk factors, signs and symptoms among community pharmacists in Ghana.

## Methods

The study objectives were addressed in a cross-sectional survey distributed to practicing community pharmacists in the Ashanti region of Ghana. The study was approved by the Committee on Human Research, Publication and Ethics, Kwame Nkrumah University of Science and Technology, Ghana (Ref: CHRPE/AP/229/17) and Biomedical Research Ethics Committee, University of KwaZulu-Natal, South Africa (Ref No: BE437/17).

### Assessment Tool

The study investigators could not find any published surveys to assess the knowledge of healthcare workers or community pharmacists on cancer, risk factors, and signs and symptoms in Ghana. However, the study investigators designed a modified questionnaire based on similar studies on breast cancer (9, 12, 13) and the American Cancer Society published list of the general signs and symptoms of cancer (14). The study questionnaires were distributed to health personnel working in the field of oncology (medical oncologist−1, radiation oncologist−1, clinical pharmacist−2, oncology nurse−1) to assess its content validity and readability. Minor modifications were made based on the response received. Pre-test was conducted on 10 community pharmacists following which some questions were modified to improve clarity. This group which participated in the pre-test were excluded from the study.

The final designed survey contained 39 close-ended questions that would be completed within an average of 15 min. The questionnaire was in four parts. The first part enclosed items that addressed community pharmacists' socio-demographics including age, gender, religion, years of experience and level of education (Table 1). Basic knowledge on cancer among community pharmacists was evaluated in the second part of the questionnaire (Table 2). Participants responded to a 15-item scale about cancer. Response ranged between “Correct,” “Incorrect,” and “I don't know.” Each “Correct” response was scored one (1) point and “Incorrect” or “I don't know” responses were both scored zero (0) point. Pharmacists with score range 0–11 were considered to have poor level of knowledge of cancer, while those with scores 12–15 were measured to have acceptable knowledge. Participant's knowledge on signs and symptoms of cancer was assessed with the third part of the questionnaires (Table 3) which contained 7-item. Scoring procedure was similar to the one used in the second part of the questionnaire. Score from 0 to 5 were measured as having poor knowledge whiles scores from 6 to 7 were regarded as having acceptable knowledge of signs and symptoms of cancer. Respondents' knowledge on causes and risk factors of cancer were evaluated using 12 true or false questions (Table 4). The scoring procedure was similar to the one used in the second part of the questionnaire. Scores between 0 and 9 were considered as having poor knowledge while scores range of 10–12 were considered as having acceptable level of knowledge of risk factors. In this study, the internal consistency reliability coefficient (Cronbach's α) calculated for knowledge items was 0.716.

**Table 1:** Sociodemographic characteristics of pharmacists (*N* = 150).

**Characteristics**	***n*** **(%)**
**GENDER**	
Male	105 (70)
Female	45 (30)
**AGE (YEARS)**	37.65 ± 10.19 (median = 36.0)
20–29	36 (24)
30–39	61 (40.7)
40–49	25 (16.7)
≥50	28 (18.6)
**RELIGION**	
Christianity	144 (96)
Muslim	6 (4)
**HIGHEST PHARMACY DEGREE**	
Undergraduate	69 (46)
Post graduate	81 (54)
**YEARS OF PRACTICE**	
1–9	81 (54)
10–19	43 (28.7)
20–29	16 (10.6)
>30	10 (6.7)
**NUMBER OF ADJUNCT STAFF**	
1	96 (64)
2	45 (30)
≥3	9 (6)
**DID YOU RECEIVE ONCOLOGY EDUCATION DURING YOUR PHARMACY PROGRAM?**	
Yes	3 (2)
No	147 (98)
**HAVE YOU EVER ATTENDED A CANCER CONTINUOUS EDUCATION BEFORE?**	
Yes	0 (0%)
No	150 (100)

*Field Data 2018. Values are expressed as n(%)*.

**Table 2:** Knowledge of cancer among participating pharmacists (*N* = 150).

	***n*** **(%)**		
**Cancer knowledge item**	**Correct**	**Incorrect**	**Don't Know**
Cancer is when abnormal cells divide in an uncontrolled way (True)	55 (36.7)	65 (43.3)	30 (20.0)
Cancer is just one disease (False)	44 (29.3)	68 (45.4)	38 (25.3)
Cancer is caused by virus (False)	26 (17.3)	78 (52.0)	46 (30.7)
Cancer cells can also spread to other parts of the body (True)	56 (37.3)	57 (38.0)	37 (24.7)
The cause of cancer is unknown (True)	50 (33.3)	71 (47.3)	29 (19.4)
Most cancers form a lump called a tumor or a growth (True)	48 (32.0)	62 (41.3)	40 (26.7)
Not all lumps are cancer (True)	54 (36.0)	74 (49.3)	22 (16.7)
Some cancers do not form tumors (True)	48 (32.0)	64 (42.7)	38 (25.3)
There are more than 100 types of cancer (True)	49 (32.7)	60 (40.0)	41 (27.3)
Cancers are usually named for the organs or tissues where the cancers form (True)	43 (28.7)	62 (41.3)	45 (30.0)
Anybody is susceptible to cancer (True)	59 (39.3)	62 (41.3)	29 (19.4)
Cancer is not life-threatening disease (False)	43 (28.7)	56 (37.3)	51 (34.0)
Cancer is an infectious disease (False)	47 (31.3)	65 (43.3)	38 (25.3)
Early detection of cancer is difficult because there are no specific symptoms (False)	48 (32.0)	60 (40.0)	42 (28.0)
The most common treatments for cancer are surgery, chemotherapy and radiation (True)	38 (25.3)	62 (41.3)	50 (33.3)

*Field Data 2018. Values are expressed as n(%)*.

**Table 3:** Knowledge of cancer signs and symptoms among participating pharmacists (*N* = 150).

	***n*** **(%)**		
**Signs and symptoms of cancer**	**Correct**	**Incorrect**	**Don't know**
Change in bowel or bladder habits is a warning sign or symptom of cancer (True)	59 (39.3)	61 (40.7)	30 (20.0)
A sore throat that does not heal is a warning sign or symptom of cancer (True)	49 (32.7)	68 (45.3)	33 (22.0)
Unusual bleeding or discharge is a warning sign or symptom of cancer (True)	55 (36.7)	67 (44.7)	28 (18.6)
Thickening or lumps in the breast or elsewhere is a warning sign or symptom of cancer (True)	91 (60.6)	59 (39.3)	–
Indigestion or difficulty in swallowing is a warning sign or symptom of cancer (True)	48 (32.0)	73 (48.7)	29 (19.3)
Obvious change in wart or mole is a warning sign or symptom of cancer (True)	56 (37.3)	77 (51.3)	17 (11.4)
Nagging cough or hoarseness is a warning sign of or symptom cancer (True)	61 (40.7)	79 (52.7)	10 (6.6)

*Field Data 2018. Values are expressed as n (%)*.

**Table 4:** Knowledge of cancer causes and risk factors among participating pharmacists (*N* = 150).

	***n*** **(%)**		
**Cancer causes and risk factors**	**Correct**	**Incorrect**	**Don't Know**
Smoking (True)	97 (64.7)	53 (35.3)	–
radiation exposure (True)	55 (36.7)	58 (38.7)	37 (24.7)
overweight and obesity (True)	40 (26.7)	64 (42.7)	51 (34.0)
Infections (True)	40 (26.7)	72 (48.0)	38 (25.3)
Insufficient physical activity (True)	46 (30.7)	70 (46.7)	34 (22.7)
Excessive alcohol intake (True)	50 (33.3)	62 (41.3)	33 (22.0)
Diet (True)	40 (26.7)	55 (36.7)	55 (36.7)
Carcinogens (True)	90 (60.0)	51 (34.0)	9 (6.0)
Hormones (True)	34 (22.7)	65 (43.3)	51 (34.0)
Chronic inflammation (True)	41 (27.3)	77 (51.3)	32 (21.3)
Excessive meat intake (True)	14 (9.3)	78 (52.0)	58 (38.7)
Lack of fruits and vegetable intake (True)	35 (23.3)	51 (34.0)	64 (42.7)

Field Data 2018. Values are expressed as n (%)

### Sample and Data Collection Procedure

Convenience sampling was used to enroll community pharmacists in Ashanti region of Ghana into the study. Trained research assistants approached study participants at the community pharmacies to administer the questionnaires and study information leaflets. Signed informed consent documents were obtained from pharmacists who accepted to fill the questionnaires.

### Analysis

Microsoft Excel, 2013 was used to enter the data and later exported to IBM SPSS statistical package (IBM Corp. Version 20.0 Armonk, NY, USA) which was then used for data analysis. Descriptive statistics were used to report study variables. Pearson's chi-square (*X*^2^) test of independence was used to test for correlations between categorical variables. Bivariate correlation analysis was done to test for correlations between continuous variables. Differences were considered to be statistically significant at *P* < 0.05.

## Results

The pilot study was conducted from November 2017 to March 2018. Of 380 questionnaires distributed to community pharmacists in Ashanti Region of Ghana as part of on-going study, the initial 150 that were completed and collected was used for this study resulting in a response rate of 39%.

### Sociodemographic Characteristics of Study Participants

The sociodemographic characteristics of study participants are summarized in Table 1.

The average age of participating pharmacists was 37.65 ± 10.19 years (median = 36.0 years) with a range from 20 to 59 years. Majority of participants were male (70%). More than half of participating pharmacists hold Bachelor's degree (BPharm) and falls within the age group of 30–39 years. Most of the participants have 1 to 9 years (54%) of working experience. Most community pharmacies have only one supporting staff (64%) in addition to the pharmacists. Almost all participants never received cancer education during their pharmacy program and never have they attended a continuous education on cancer.

### Knowledge of Community Pharmacists on Cancer

Table 2 shows the responses of community pharmacists toward a list of statements on cancer. More than half of the participants (52%) acknowledged that the statement, cancer is caused by a virus, is incorrect. Close to half of the participants (49.3%) assent to the fact that not all lumps are cancer is incorrect. The item, anybody is susceptible to cancer, was admitted by only 39.3% as a correct statement. In order to assess the level of knowledge on cancer, responses were evaluated in the form of scores out of 15 points. Mean score was 7.2 ± 1.71 out of a maximum score of 15 points (median = 8, range = 1–15) categorizing the knowledge on cancer as poor. The overall assessment of pharmacists' knowledge revealed that 76.7% of them had poor level of knowledge on cancer.

### Knowledge of Community Pharmacists on Signs and Symptoms of Cancer

Table 3 shows the responses of community pharmacists toward a list of warning signs and symptom of cancer. Over half of participants (52.7%) recognized that the statement, nagging cough or hoarseness is incorrect as a sign and symptom of cancer. The statement, obvious change in wart or mole is a warning sign or symptom of cancer was agreed by more than half of participants (51.3%) as incorrect statement. Few of the participants (32%), agreed that a sore throat that does not heal is a warning sign or symptom of cancer. Overall, 82% of participants had poor knowledge on this section.

In order to evaluate the knowledge of pharmacists on signs and symptoms of cancer, responses were assessed in form of scores out of 7 points. Mean score was 3.65 ± 1.82 out of a maximum score of 7 points (median = 4, range = 1–7) categorizing knowledge on cancer signs and symptoms as poor. The overall evaluation indicates that 82% of pharmacists had poor knowledge on cancer signs and symptoms.

### Knowledge of Community Pharmacists on Causes and Risk Factors of Cancer

Table 4 shows the responses of community pharmacists toward a list of cancer risk factors.

Most participants were able to recognize smoking (64.7%) and carcinogen (60%) as a cause or predisposing factor for cancer. More than half of participants assent to the fact that chronic inflammation (51.3%) and excessive meat intake (52%) are incorrect as a cause and risk factors for cancer.

Knowledge of participants on causes and risk factors of cancer was evaluated by assessing the response in the form of scores out of 7 points. Mean score was 5.65 ± 1.12 out of a maximum score of 12 points (median = 4, range = 1–12) categorizing participants knowledge on causes and risk factors of cancer as poor. The overall assessment of the knowledge score indicates that 65.3% of pharmacists had poor knowledge regarding causes and risk factors of cancer.

### Relationship of Demographics and Characteristics of Pharmacy Practice With Knowledge on Cancer, Signs and Symptoms of Cancer, Causes, and Risk Factors of Cancer

Bivariate correlation analysis of continuous variables is shown in Table 5. Age has no significant correlation with scores of general knowledge on cancer as well as causes and risk factors of the disease among participating community pharmacists (*r* = −0.026, *P* = 0.316 and *r* = −0.015, *P* = 0.381, respectively). However, an inverse significant correlation was found between age and knowledge scores of signs and symptoms of cancer (*r* = −0.087, *P* = 0.031).

**Table 5:** Correlation analysis of continuous variables (*N* = 150).

**Mean age of pharmacists**	**Knowledge on cancer**			**Knowledge on signs and symptoms**			**Knowledge on causes and risk factors**		
	**Mean score**	***r***	***p***	**Mean score**	***r***	***p***	**Mean score**	***r***	***p***
37.65 years	7.2	−0.026	0.316	3.65	−0.087	0.031	5.65	−0.015	0.381

A chi-square test of independence was performed to examine the relation between sociodemographic characteristics of pharmacists and their categorical level of knowledge on cancer (poor vs. acceptable). Overall, no significant associations were found between sociodemographic variables for community pharmacists and knowledge of cancer, signs and symptoms, or causes and risk factors. Gender (*X*^2^ = 24.14, *p* = 0.06), years of practice (*X*^2^ = 28.32, *p* = 0.06), and level of education (*X*^2^ = 33.10, *p* = 0.08) were not associated with cancer knowledge of community pharmacists in this study.

## Discussion

Key findings from this study indicates that most community pharmacists have poor knowledge on cancer, signs and symptoms, causes and risk factors of cancer.

Similar study on the involvement of community pharmacists in breast cancer health promotion in United Arab Emirate, also indicated insufficient knowledge in breast cancer (15). A survey of community pharmacists on breast cancer health promotion in Qatar also indicated low breast cancer knowledge (10). However, findings gathered in a study which assessed knowledge, attitudes and barriers toward breast cancer health education among Jordanian community pharmacists indicated an acceptable level of knowledge among them (13).

From the main researcher experience as an adjunct faculty member in one of the pharmacy school in Ghana, cancer diseases are not part of the clinical courses of the undergraduate pharmacy curriculum. It only forms part of the post graduate clinical course. Recently with the introduction of Doctor of Pharmacy (PharmD), oncology course is part of the curriculum. But graduates of this program were not included in this study because the data was collected before graduation of the first batch. Moreover, not all pharmacy schools in the country are offering this new PharmD programme, hence it is also necessary to revise and include oncology education in undergraduate pharmacy curriculums to improve their knowledge on cancer before coming practicing pharmacists.

All the participants acknowledge that they have never attended cancer continuous education before. Also, to the best of our knowledge, continuous pharmacy education on cancer has never been offered in Ghana before, hence knowledge in cancer is very lacking and this might have reflected on the results obtained.

In recent years, pharmacy practice has shifted to the provision of patient-oriented pharmaceutical service. With increase in the burden of the disease in the country, community pharmacists must improve upon their knowledge on cancer to be able to play the evolving professional role in the healthcare system. Therefore, there is an urgent need for continuing education programmes structured for community-based pharmacists in order to be well-prepared to provide effective cancer health education.

The knowledge level was found to be poor in male pharmacists than in female pharmacists. The distribution of male (70%) to female (30%) pharmacist population for this pilot was not equal and may have accounted for the result obtained. Reports from other studies done in different population in developing countries on level of knowledge on breast cancer indicated low level of knowledge among females (16–22). These were attributed to low levels of education. Even though all studies reported were done in breast cancer, findings cannot be generalized but attributing factors for findings can be generalized.

Evidence from this pilot study also indicates that there is a low level of knowledge of cancer warning signs and symptoms among community pharmacists. The most correctly identified warning sign was “thickening or lumps in the breast or elsewhere.” A study done in Jordan among community pharmacists indicated this warning sign and symptom as the most identified, even though the study was on breast cancer (13). These findings can be attributed to on-going advocacy on breast cancer worldwide (23). Another study which was done in the general public but not with healthcare professionals or community pharmacists also had majority of the general public been able to identify this warning sign. This by them was attributed to high profile and on-going media campaigns for testicular and breast cancer (24).

In Ghana, breast cancer is the most advocated cancer. Television and radio campaigns focuses on identification of lump in the breast as a major sign of breast cancer. Religious groups, professional groups, various societies, etc. in the country organize talks for women with focus on identification of lumps as a warning sign of breast cancer. Hence lump in the breast as a sign of breast cancer is well-known in Ghana. This might have accounted for “lump in the” breast being the most identified warning sign of cancer. This implies that community pharmacists can use media education or public talk to promote cancer health if they have enough knowledge on signs and symptoms of cancer.

Poorly recognized sign and symptom was “indigestion or difficulty in swallowing.” To the best of our knowledge, no studies have been done in any healthcare professional group or this group to assess the level of knowledge on this cancer sign and symptom. However, a study done in Britain on the public awareness of warning signs for cancer (25) reported that this sign was poorly recognized. Keeney et al., in his study, further explained that the level of recognition of this sign did not improve among this population after years of intervention before his study (24). Another work done in Britain on public awareness of cancer, a population-based survey of adults also indicated poor recognition of this sign (26). Though these studies were done in the general public and not in health professionals or community pharmacists, further exploration of the reasons may be required.

Women are expected to have more experience than men in the area of health, at least partly because of the role many Ghanaian women play as family care-providers in our culture. It was therefore anticipated that categorical analysis of the data will show an association between gender and level of knowledge of cancer among community pharmacists. However, findings in this study showed no significant difference in knowledge among male and female pharmacists.

Overall awareness on risk factors of cancer was poor among community pharmacists. Knowledge among the study participants showed larger emphasis on smoking, carcinogen and radiation as risk factors. A study which evaluated the knowledge level of community pharmacists on risk factors of breast cancer showed larger emphasis on hereditable (family history) and genetic (genetic factors) causes (13). Another study done in United Arab Emirate, which measured the knowledge level of community pharmacists on risk factors of breast cancer, indicated a high response for “use of hormone replacement therapy” as a risk factor for developing breast cancer (15). A study done to assess the knowledge, perception, practice and barriers of breast cancer health promotion activities among community pharmacists in two districts of Selangor state, Malaysia, indicate high recognition of excessive alcohol consumption and early mensuration or late menopause as risk factors (9). Different risk factors are identified in various studies, and this is due to different factors used in the questionnaires by the various researchers. For this pilot study, smoking and carcinogen were the most recognized risk factors based on the items on the questionnaire.

Smoking is well-known risk factor for many diseases in Ghana. From basic pharmacology, carcinogen is well-known to be a cancer-causing agent. This might explain why it was chosen by many participants. This also implies that education of community pharmacists on risk factors of cancer is necessary to empower them to undertake cancer health promotion activities.

In summary, findings of the study indicate that community pharmacists have poor knowledge on cancer as a disease. Also, their knowledge on signs and symptoms of cancer and risk factors of the disease was poor.

## Limitation

We acknowledge that our study has few limitations. The use of convenience sample in the study could create a selection bias which can affect the results obtained. Our study was also limited by its small sample size and low response rate. Furthermore, male pharmacists were over-represented and pharmacists with over 20 years of experience underrepresented in our preliminary data. Retrieval of questionnaires from community pharmacists was difficult. This may raise concern about reliability of their responses. Also, self-report survey that was used rely on participants completing the questionnaire themselves and truthfully. This cannot be ascertained in this study and hence can affect the results obtained.

## Conclusion

This pilot study is the first known attempt to assess community pharmacists' level of knowledge on cancer, its signs and symptoms, its causes and risk factors in Ghana. Despite its limitations, this pilot study provided valuable data, on which further data can be built. Findings from the study suggested poor level of knowledge on cancer, signs and symptoms, causes and risk factors among community pharmacists in Ghana. This can be attributed to knowledge gap. This finding has important implication for developing formal undergraduate training programs and promoting the pursuit of continuous pharmacy education on cancer programs to improve the knowledge of community pharmacists in Ghana.

The survey instrument was standardized before administering to the study participants. It also provided the participants with a standardized stimulus. With such high reliability obtained, the researcher's own biases were eliminated. The findings obtained from the study agree with findings of other studies conducted in this area. This gives credence to the survey instrument and findings of the study. Results obtained further indicate that the questions were adequate to assess the knowledge level of community pharmacists in Ghana on cancer.

Data obtained from this study indicated a viability and need of conducting a full-scale research in this workforce even though few questions were not answered. Full scale study will be necessary to get a better assessment of the level of knowledge of community pharmacists on cancer in Ghana.

## Data Availability Statement

The datasets generated during the study are available in the fig share repository, https://figshare.com/s/6916eaccd3b002a45df1

## Author Contributions

All authors have contributed significantly to the intellectual content of this study. KM, FO, and VB have substantially contributed to the conception of the research idea, design of the study, and collection, analyses, and interpretation of data, and writing and/or revising the manuscript. All authors have read and approved the final version of this manuscript.

### Conflict of Interest Statement

The authors declare that the research was conducted in the absence of any commercial or financial relationships that could be construed as a potential conflict of interest.
